# A novel trust-scoring system using trustability co-efficient of variation for identification of secure agent platforms

**DOI:** 10.1371/journal.pone.0201600

**Published:** 2018-08-02

**Authors:** Adri Jovin John Joseph, Marikkannan Mariappan

**Affiliations:** 1 Department of Information Technology, Sri Ramakrishna Institute of Technology, Coimbatore, Tamil Nadu, India; 2 Department of Computer Science and Engineering, Institute of Road and Transport Technology, Erode, Tamil Nadu, India; Victoria University, AUSTRALIA

## Abstract

Mobile Agent systems are prevalent in Distributed Environment due to the autonomy and adaptability in diversified situations. Mobile Agents are capable of movement from one platform to another and hence it is a need to ensure the safety of the Mobile Agent during the phase of transit. Since Mobile Agents are capable of deciding their itinerary dynamically, a decision support system which helps to ensure the trustworthiness of a platform would complement the decision. A Trust Scoring System is therefore proposed to measure the trustworthiness of a platform based on a metric termed Trust Score. Trust Score varies dynamically with respect to time and is based on a function comprising of five parameters namely Persistence, Competence, Reputation, Credibility and Integrity. In order to reduce the computational latency, another metric named Trust Rank for platforms, based on Trustability co-efficient of variation is introduced. The experimentation is done in cloud environment with platforms located at different geographic regions. The performance is evaluated in terms of response time and accuracy of decision. From the experimental results, it is evident that the Trust Ranking mechanism consumes less response time and improves the accuracy of decision of Mobile Agents during their itineraries, compared to that of the prototype system that uses Trust Score alone as a measure of decision-making.

## Introduction

Mobile Agents are Software Agents which possess the capability of mobility. Mobile Agents are capable of providing promising solutions to a number of issues associated with E-commerce, Network Management, Security, Database Access etc. A Mobile Agent is characterised by three parts namely the Code, State and Data [1]. The movement of the Mobile Agent is termed migration and the travel plan thus executed is termed itinerary. A Mobile Agent requires an execution Platform to get instantiated, migrate and get executed. The Platform contains all the resources required for the Mobile Agent. A Mobile Agent moves from one platform to another in order to collect and to process data. This migration is generally termed weak mobility. If the migration involves mobility of state along with the data, then it is termed strong mobility[2].

The execution Platform plays a significant role in a Mobile Agent System. Some platforms are capable of providing the data whereas some other are capable of processing the data. For convenience, platforms are termed data platforms, which make data requests and process the data and server platforms, which serve as data repositories. Apart from processing, the data platform stores the results of data acquired from the server platforms. Since the platform is a major component in the Mobile Agent system, it is required to assure the safety of the platform. The platform is the one that is responsible for the processing of data carried by the Mobile Agents. One should be aware of the fact that the same type of service may be provided by multiple platforms in a distributed system based on affinity and convenience of service.

A number of literature provide evidence that the platforms may be compromised, which in turn affects the Mobile Agents. When a platform is adversely affected by a malicious code, it consequently attacks a Mobile Agent. This is termed Platform-to-Agent attack[3]. The Mobile Agent subsequently attacks the other Platforms and other Agents in the system.

Selecting the appropriate platform for execution plays an important role in the proper function of the Mobile Agent Environment. In Mobile Agent systems, the itinerary is either decided statically or dynamically. Before setting an itinerary, selection of platform is important. The design of a decision support system will be a suitable solution for the problem of itinerary planning. There are various parameters related to the selection of a platform. This work introduces Trust as a parameter for selecting a Platform and Trust Score as a measure for assessing the trustworthiness of a Platform. Based on the Trust Score, the Mobile Agent chooses the execution platform.

The paper is organised as follows: Section 2 discusses the existing work related to the selection of platforms based on trust. Section 3 introduces the new parameter used for the calculation of trust namely Trust Score and discusses the parameters that contribute to the calculation of Trust Score. The calculation of the Trustablility co-efficient of variation, using which the platforms are ranked is further discussed in this section. Section 4 discusses the results obtained by the experimentation and the analysis of the performance, in terms of response time and accuracy of decision. Section 5 concludes the research contribution of this work.

## Related works

A few work related to the selection of a platform, trust etc. is studied to proceed with this work.

Bamasak and Zhang [4] introduced the use of Trusted Third Party for managing the reputation of a host. This involves the TTP-hosts Subgroup Selection (TSS) algorithm, which helps in identification of the group of TTP-hosts taken into account their trust values. The Trust here, is calculated on the basis of user input and rating. But, recent studies make it quite clear that Trust is not just a parameter based on ratings. It contains a number of parameters. Trust and Reliability Updating algorithm follow a process in which the owner of the Mobile Agent is offered with the provision to rate the TTP-host subgroup. The rating in turn is considered as trust. The ratings can be considered as reputation of the subgroup of TTP-host and cannot be considered as trust. It is possible that a malicious set of TTP-host may gain a high reputation by providing satisfactory services to the Agent owner during the initial phases of the transaction. Later on, it may start attacking the agents.

Wang et al. [5] introduces the concept of trust entropy to measure the degree of trust. A trust model is proposed in this system which is regarded as one of the most preliminary and classical method of measuring trust related to a Mobile Agent System. It involves trust formation, dissemination and evolution. In the formation phase, the data required to calculate the trust are collected. This introduces the basis of Trust Score by means of certain entropy based calculations. Being an elementary idea, this work considers only security and malicious result comparison for the calculation of Trust Score. However, just two parameters are not sufficient to measure trust, since trust is a combination of a number of parameters. This can be visualised from the work described below.

Vijay Varadharajan[6] pioneers the deployment of trust based computing in Mobile Agent Systems. In his work, he describes trust as a combination of basic security services namely authentication, integrity, confidentiality and access control. Mobile Agents carry a passport which is the trust authenticating factor. This passport, on acceptance by the platform approves the access towards all the resources in the platform, by the Mobile Agent.

Bedi and Gaur[7] propose a methodology of evaluation of trust by considering the quality attributes related to Multi-agent systems. They state that this model encourages System Developers to develop systems by including all the quality parameters which in turn have a better impact over the Software Development Life Cycle also. The key quality parameters are taken from the stakeholders. The researchers use Intuitionistic Fuzzy Sets to evaluate the effective contribution of quality over trust. The quality parameters, when left with the choice of the stakeholders will not only be confined with the technical parameters but also the non-functional parameters. Therefore, the non-functional parameters need to be correlated with measurable parameters to proceed with this method.

Shen and Wu [8] introduces a Mobile Agent Security Supporting Environment using a combination of Trusted Software Stack and Trusted Computing Platform. This work utilizes the recommendations of the Trusted Computing Group to improve the security of the Mobile Agent System. The Trusted Computing Platform contains a number of tools and protocols to protect the Mobile Agents from being attacked. This work introduces the measurement of security parameters such as integrity, confidentiality etc. and some preliminary ideas on credit measurement and communication methodologies between the Mobile Agents and platforms.

Ye et al.[9] considers trust as a parameter involving social aspects, direct observations and past experience. It involves collective parameters, in addition to the parameters mentioned by Wang et al.[5]. This work gives a formal definition for trust and evaluates trust based on formal methods.

Zuo and Liu[10] used opinion-based belief structure to interpret, cumulate and to calculate the reputation of a host based on seven parameters namely Information Accuracy, Purpose, Currency, Authority, Information Service User friendliness, Appropriate Technology and System integrity. An opinion-based belief structure is formulated utilizing the above mentioned parameters. The belief structure ranks the hosts taken into account their performance with respect to the above mentioned parameters. Mobile Agents rely on the hosts with better ranking for services and avoid hosts with least performance.

Pouryazdan et al.[11] used a concept of User Reputation Score to solve certain problems related to Mobile Crowd-sensing for mobile applications. User Reputation Score evidently provides promising results in extending crowd sensing to mobile applications.

Shehada et al.[12] proposed a Trust-based Secure Mobile Agent Protocol which is used in Mobile Agent transactions for Vehicular Communication systems. This protocol ensures protection to the Mobile Agents from numerous types of attacks such as Man in the middle attack, replay, masquerade, modification, and unauthorized attacks. It is a simulation model and hence the certainty of the protocol cannot be determined in real-time.

There are a very few work related to the act of recommending a Mobile Agent to visit a specific host or a platform. The major challenge in decision support systems is the selection of the parameters required to measure the performance of a host or a platform. Apart from the parameters mentioned by Zuo and Liu [10], factors such as interoperability, ability to synchronize etc. have to be taken into consideration due to the diversified categories of platforms and compatibility of a Mobile Agent with a platform. This work is an extract of an extensive analysis over the various physical and logical parameters related to performance and trust. Steps are taken to represent the parameters in terms of technical parameters.

Concepts of Trust and Reputation score, along with managerial principles forms the basis of the proposed work. The proposed work quantifies the managerial principles based on different parameters and converge them to a simple metric, namely Trust Score. Trust Score is further processed to derive the Trustability co-efficient of variation, which helps in providing rank to the platforms.

## Trust Scoring

A Mobile Agent readily interacts with any platform which is considered trustworthy. Trustworthiness or Trust is a broad term which is so hard to describe in the domain of technology. It is not simply a term concerned with security alone. It is a relative measure. This work proposes an elementary model of Trust Scoring which measures the trustworthiness of a platform to earmark it a trustable source for interaction.

Based on previous studies and inferences, trust can be defined as a summative function of Persistence (P), Competence ($C_{p}$), Reputation ($R_{e}$), Credibility ($C_{r}$) and Integrity ($I_{g}$). Being an uncomplicated mechanism, a simple model for measuring trust is proposed in this system.

The Trust Score can be calculated using the Eq (1)

Trust Score

$$\text{Trust}\;\text{Score}\;T=F\left(P,,,C_{p},,,R_{e},,,C_{r},,,I_{g}\right)=K.\left(P,+,R_{e},+,C_{r},+,I_{g},+,C_{p}\right)$$ (1)

where K is the Trust Equating Factor or Trust Normalization Constant.

The value of Trust score lies between 0 and 1. Each parameter to measure trust is normalised to unit value. The value of Trust Normalization Constant is 0.2 since all the parameters are equally weighted.

### Persistence

Persistence is measured through the stability and consistency of the platform.

$$\text{Persistence}\;P=F\left(S,,,C_{s}\right)$$

Stability (S) refers to the ability of the platform to cater an appropriate resource or data for an appropriate request made by the Mobile Agent. Suppose the resource/data is denied by the platform m times for the request made by n Mobile Agents, then Stability can be calculated as

$$S=\left(n,-,m\right)/n$$

Consistency ($C_{s}$) is the ability of the platform to respond whenever it receives a request from the Mobile Agent with the possible resource, in an allowed way. Consider n number of Mobile Agents requesting for a resource on a platform. If the platform denies the resource in the allowed way or a legitimate request, it is considered a failure. Whereas, if the resource is denied in a way that is not allowed or a denial for an illegitimate request, it is considered successful. Consider the denial of access towards m request out of the n requests made by Mobile Agents, then the Consistency can be calculated as

$$C_{s}=\left(n,-,m\right)/n$$

$$P=F\left(S,,,C_{s}\right)=\left(S,+,C_{s}\right)/2$$

### Competence

Competence is measured using the average response time and average turn-around time taken by the platform for processing the requests.

$$C_{p}=F\left(T_{T},,,T_{R}\right)$$

The total time taken from the submission of the request to the completion of the task by the platform to process the request placed by the Mobile Agent is termed turn-around time.

Suppose, there are n Mobile Agents. Each of them places the request to the platform and the tasks get completed after time$t_{t}$. Then, the average turn-around ($T_{T}$) will be calculated as

$$T_{T}=t_{t}/n$$

Response time is the time taken from the submission of a request by the Mobile Agent to the time taken by the platform to make its first response. Consider there are n Mobile Agents. Each of them consumes a response time r and let the total response time be $t_{r}$. The average response time ($T_{R}$) will be calculated as

$$T_{r}=t_{r}/n$$

Let $T_{ST}$ be the Standard Turn-around Time and let $T_{RT}$ be the Standard Response Time. Then Competence is calculated as

$$C_{p}=F\left(T_{T},,,T_{R}\right)=\left(\left(T_{ST},/,T_{T}\right),+,\left(T_{RT},/,T_{R}\right)\right)/2$$

### Reputation

Reputation is a cumulative measure of availability of the information and the degree of compatibility of the platform with other agents. Let I be the Information Availability and C be the Compatibility measure, then

$$R_{e}=F\left(I,,,C\right)$$

Information Availability (I) is the tendency of the platform to respond towards the request of a Mobile Agent. Suppose a Mobile Agent sends a request to a platform, the platforms respond to the request, then the request has been successfully responded. If there is no response, then it considered a failure. Consider n Mobile Agents send a request to the platform of which m requests are not responded, then the Information availability I can be measured as

$$I=\left(n,-,m\right)/n$$

Compatibility (C) is the ability of the platform to synchronize with the Mobile Agent. Consider n number of Mobile Agents try to communicate with the platform and m Mobile Agents cannot get synchronised with the platform. The compatibility C can be measured as

$$C=\left(n,-,m\right)/n$$

$$R_{e}=F\left(I,,,C\right)=\left(I,+,C\right)/2$$

### Credibility

Credibility $C_{r}$ is the measure of accuracy and effectiveness of the results produced by the platform to the Mobile Agents.

$$C_{r}=F\left(A,,,E\right)$$

Accuracy (A) is defined as the degree of correctness of the data provided by the platform. This can be verified by the acceptance of the data with other platforms when used by the Mobile Agent for further processing. If there are m number of data out of ndata, which are not accepted by other platforms for processing, then the accuracy A is measured as

$$A=\left(n,-,m\right)/n$$

Effectiveness (E) is defined as the usability of the data provided by the platform to the Mobile Agent by other platforms. Suppose, the data provided by a platform did not produce expected results for m processes out of n processes, in other platforms, then the effectiveness E is measured as

$$E=\left(n,-,m\right)/n$$

$$C_{r}=F\left(A,,,E\right)=\left(A,+,E\right)/2$$

### Integrity

Integrity is calculated by the degree of availability and confidentiality of the platform.

$$\text{Integrity}\;I_{g}=F\left(A,,,C_{f}\right)$$

Confidentiality ($C_{f}$) is the measure of non-disclosure of the information to other parties except between the sender and receiver. In a best case, it shall be assumed that the information is not disclosed among the other members. In case if m breaches were found in the transaction of the platform with m Mobile Agents, then the confidentiality $C_{f}$ is measured as

$$C_{f}=\left(n,-,m\right)/n$$

$$I_{g}=F\left(A,,,C_{f}\right)=\left(A,+,C_{f}\right)/2$$

The parameters cumulatively contribute to the Trust Score of the platform.

### Trust Lookup Table

**Fig 1:**
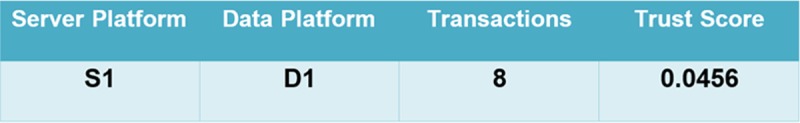
Trust Lookup Table.

Trust Lookup Table is a tabular arrangement of the Trust Score which contains four fields namely server platform, data platform, number of transaction and Trust Score. The total number of transactions out of which the Trust Score is attained is also stored. Each entry of Trust Score is the Trust Score of a server platform with respect to the transactions made by the Mobile Agents generated from the data server. For each transaction made by the Mobile Agent with a server platform, the Trust Score is updated. Fig 1 shows the typical structure of a Trust Lookup Table.

Trust Score specified in this table is the Trust Score of the server platform $S_{1}$ with respect to the data platform $D_{1}$. Similarly, the Trust Score for each server platform with respect to a data platform is updated for each transaction in the system. Consider, there are *m* number of data platforms and *n* number of server platforms. Trust Lookup Table will contain a maximum of m×n Trust Score entries.

### Trust Score based Itinerary Planning Algorithm

Mobile Agents follow the Trust Score based Itinerary Planning Algorithm to propagate from one host to another. Itinerary, as discussed in earlier chapters is a travel plan made by the Mobile Agent. The itinerary is decided either at the time of origin by the originating host or by the Mobile Agent itself during the phase of the travel.

Trust Score based Itinerary Planning uses the Trust Score as a parameter to decide the itinerary. If the Trust Score is high for a server platform, then the server platform is more trustable.

**Fig 2:**
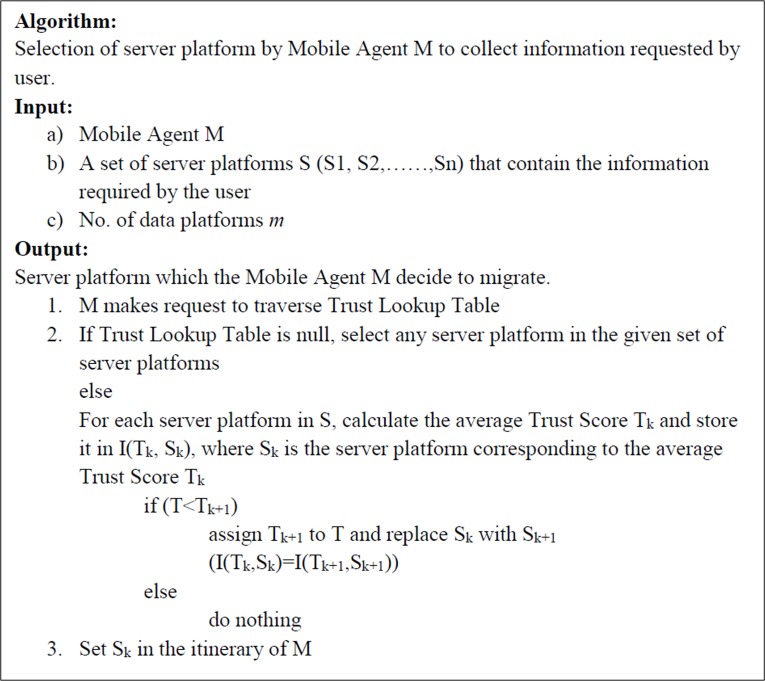
Trust Score based Itinerary planning Algorithm.

Trust Score based Itinerary planning algorithm is shown in Fig 2.

It is observed that the Mobile Agent or the data platform need to make a linear search in the Trust Lookup Table to find the mean Trust Score of a server platform. Consider there are *m* data platforms and *n* server platforms. In this case, the maximum number of entries in the Trust Lookup Table will be m×n. The data platform or the Mobile Agent need to collect the details pertaining to every server platform, calculate the mean Trust Score, compare and then decide which server platform to visit, based on the mean Trust Score. This adds overhead to the Mobile Agent or the data platform in decision making. This needs to be reduced to achieve better results.

### Trustability co-efficient of variation

In case of the Trust Scoring system, the Trust Lookup Table maintains the Trust Score entries of each server platform with respect to the data platforms, available in the system. As mentioned earlier, if there are m data platforms and n server platforms, the Mobile Agent or the data platform needs to traverse through m×n entries in the Trust Lookup Table to make the decision. It is observed that the function of the Trust Monitor is to maintain the Trust Lookup Table alone.

The efficiency of the system can be increased if the overhead of calculation of Mean Trust is decreased from the Mobile Agent. The responsibility can be transferred to the Trust Monitor. It is proposed that the server platforms can be ranked based on the trustworthiness. In a statistical perspective, the more is the number of samples, the more accurate will be the inference. Therefore, in a Mobile Agent System, the more is the number of transactions, the more will be the accuracy in decision making based on the trustworthiness. In this view, considering the mean of Trust Score may not provide an amicable solution in decision making. Hence the co-efficient of variation is computed for each server platform. This unifies the entries pertaining to a server platform into a single value. The measure here, is termed the Trustability Co-efficient of Variation (*V*) which is computed using Eq (2).

$$V=\frac{\sigma_{T}}{M\left(T\right)}\times 100$$ (2)

where

$$M\left(T\right)=\frac{\overset{n}{\underset{k=1}{\Sigma }}T\left(k\right)}{n}$$

and

$$\sigma_{T}=\frac{\overset{n}{\underset{k=1}{\Sigma }}\left(T,\left(k\right),-,M,\left(T\right)\right)}{n}$$

where *T(k)* is the Trust Score of server platform with respect to the *k*^*th*^ data platform and n is the total number of data platforms with which the server platform is associated.

The Trustability Co-efficient of Variation (*V*) is collected for all the server platforms in the system and is maintained in the Trust Rank Table, a derivative of the Trust Lookup Table, in the Trust Monitor. The Trustability Co-efficient of Variation is inversely proportional to the trustworthiness of the server platform. If the value of Trustability Co-efficient of Variation is minimal for a server platform, then the server platform is more trustworthy.

### Trust Rank Table

**Fig 3:**
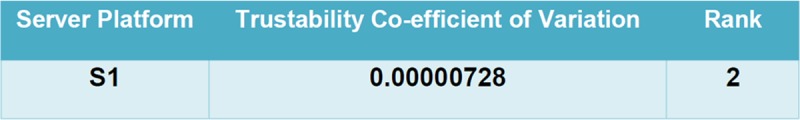
Trust Rank Table.

This is an additional table maintained by the Trust Monitor along with the Trust Lookup Table. Trust Rank Table stores the Trustability Co-efficient of Variation which contains three fields namely server platform, Trustability Co-efficient of Variation and Rank. If the Trustability Co-efficient of Variation is low, then the rank will be brought up. Fig 3 illustrates the structure of a Trust Rank Table.

For each transaction happening within the Mobile Agent System, the Trust Rank table is refreshed by the Trust Monitor. For a Mobile Agent System comprising of *n* server platforms and *m* data platform, there will be *m* entries in the Trust Rank Table.

Therefore, the introduction of Trust Rank Table reduces the time taken for decision making *m* time compared to that of the time taken for decision making in a system which is based on Trust Score alone. This is detailed in the further sections through experimental inferences. For convenience, this system is termed ‘Trust Ranking System’.

## Results and discussion

### Experimental setup

**Fig 4:**
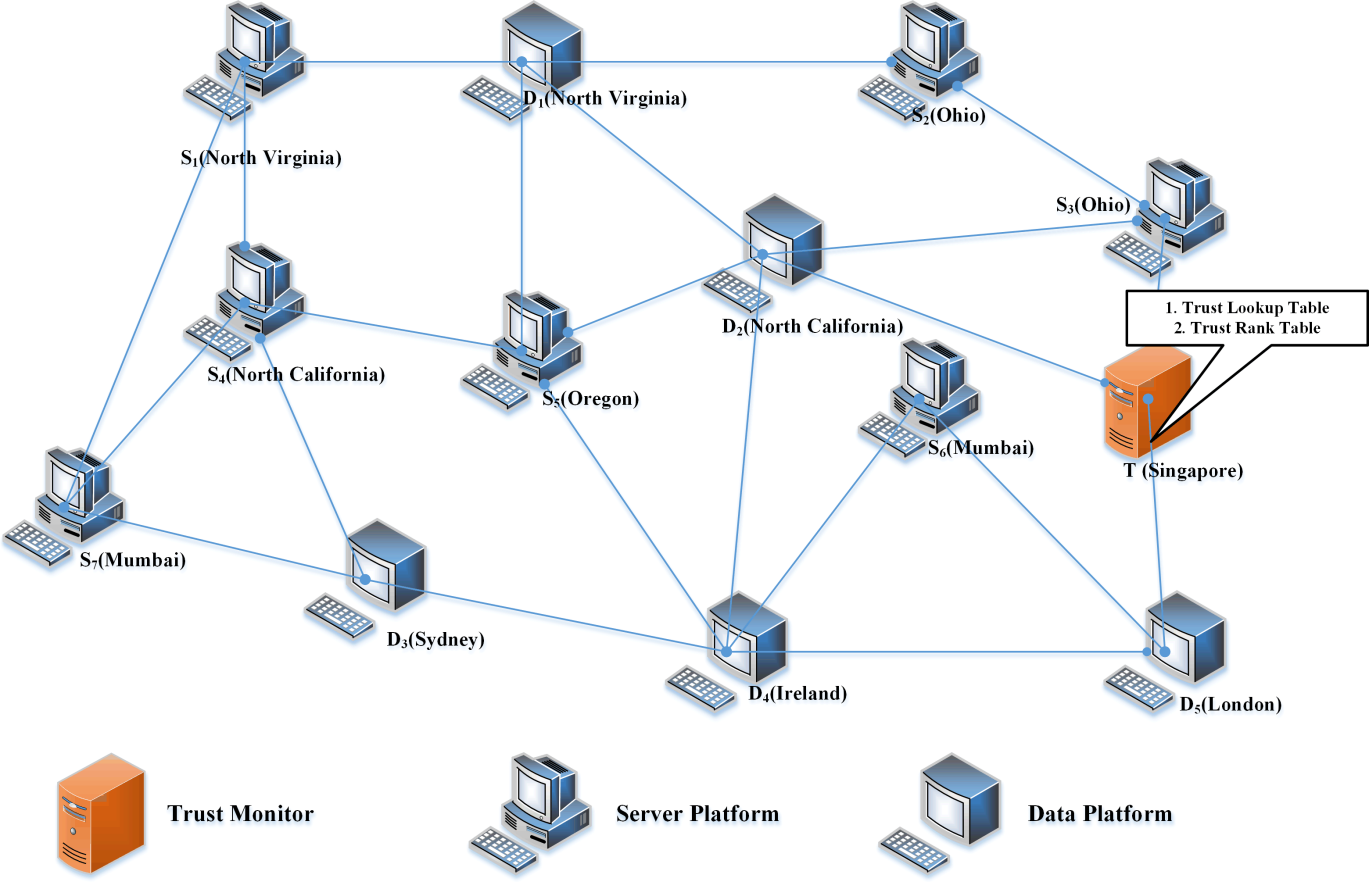
Experimental setup.

A typical illustration of the experimental setup is as shown below in Fig 4. The experimental setup comprises of seven server platforms (*S*_*1*_, *S*_*2*_, *S*_*3*_, *S*_*4*_, *S*_*5*_, *S*_*6*_, and *S*_*7*_) and five data platforms (*D*_*1*_, *D*_*2*_, *D*_*3*_, *D*_*4*_, *D*_*5*_). Server platforms and data platforms are configured as cloud instances using the Amazon Web Services. Tamil Glossary Data Set provided with S1 Appendix is used for the experimentation. Each category of the glossary data is stored in each server platform. Some of the server platforms may contain more than one category of data. The datum is requested by the data platforms when a user posts a query to the data platform.

In order to assess the performance of the system in a real-time mode, the instances are configured at different locations. The locations of the server platforms are as follows:

*S*_*1*_ - North Virginia
*S*_*2*_ - Ohio
*S*_*3*_ - Ohio
*S*_*4*_ - North California
*S*_*5*_ - Oregon
*S*_*6*_ - Mumbai
*S*_*7*_ - Mumbai

The locations of the data platforms are as follows:

*D*_*1*_ - North Virginia
*D*_*2*_ - North California
*D*_*3*_ - Sydney
*D*_*4*_ - Ireland
*D*_*5*_ - London

Trust Monitor is located at Singapore.

Each instance is configured to be a storage instance using the Amazon Elastic Compute Cloud (EC2)[13]. The storage instance in turn is connected to the database instance which is configured using the Amazon Relational Database Service (RDS)[14] through the Amazon Route 53 Domain Name System[15].

Under EC2, the service namely C1 High CPU (c1.medium) is utilised in all the data and server platforms. The c1.medium has the following configuration:

Memory - 1.7 GiB
Processor - Intel Xeon E5–2670
vCPUs - 2 CPUs
Storage - 350 GiB HDD with 900 MB swap + 1 x 4 SSD
Architecture - 64-bit

All the server platforms and data platforms are associated with the database instance service namely DB M3 (db.m3.medium). Each db.m3.medium instance possesses the following configuration:

Memory - 3.75 GiB
Storage - 1 x 4 SSD
Processor - Intel Xeon E5–2670
vCPUs - 1 CPU
Architecture - 64-bit

Each EC2 instance is installed with a distro of Linux, CentOS [16] as the operating system. The IBM Aglets Software Development Kit 2.0.2 (ASDK) [17] is deployed to develop Mobile Agents. Since the mobility of the state is not a major concern, IBM ASDK is used. IBM ASDK supports weak mobility, in which the code and data alone gets transferred from one host to another. Each instance is considered a host. Since ASDK is a Java based development environment, OpenJDK is installed for platform support.

### Experimental results

Data platforms possess a user interface through which a user can interact with the system and initiate requests. The data platform generates a Mobile Agent for each request. Mobile Agents originated by the data platform travel to the server platforms to collect data. The datum is either processed by the Mobile Agent or the data platforms. Data platforms process the data collected by the Mobile Agents once the Mobile Agents return back to it.

At the commencement of each transaction, the Trust Lookup Table is verified for the Trust Score of each server platform by the data platform, based on which the itinerary of the Mobile Agent generated by the data platform is decided. Trust Score of each server platform with respect to a data platform is stored in the Trust Lookup Table available in the Trust Monitor. The Trust Lookup table contains three fields namely server platform, data platform and trust score. Initially, Trust Score is zero for all platforms.

A snapshot of the Trust Lookup Table is shown in Table 1. It is observed that the Trust Score of each server platform with respect to a data platform is recorded in the Trust Lookup Table. Therefore, the Trust Score with reference to each data platform, for a server platform is variable and is dependent on the experience of the Mobile Agent. However, all these Trust Score values have to be evaluated by a data platform or a Mobile Agent to set its itinerary. Therefore, the mean Trust Score is considered as a parameter for planning the itinerary and migration.

**Table 1:** Trust Lookup Table.

Server Platform	Data Platform	Transactions	Trust Score
*S*_*1*_	*D*_*1*_	5	0.7971
*S*_*2*_	*D*_*1*_	6	0.7569
*S*_*3*_	*D*_*1*_	8	0.79167
*S*_*4*_	*D*_*1*_	9	0.77222
*S*_*5*_	*D*_*1*_	8	0.87599
*S*_*6*_	*D*_*1*_	4	0.525
*S*_*7*_	*D*_*1*_	4	0.28452
*S*_*1*_	*D*_*2*_	5	0.78766
*S*_*3*_	*D*_*2*_	9	0.83833
*S*_*1*_	*D*_*3*_	10	0.82444
*S*_*4*_	*D*_*3*_	9	0.76794
*S*_*1*_	*D*_*4*_	5	0.7971
*S*_*2*_	*D*_*4*_	6	0.65274
*S*_*3*_	*D*_*4*_	8	0.79167
*S*_*4*_	*D*_*4*_	9	0.77222
*S*_*5*_	*D*_*4*_	8	0.76627
*S*_*6*_	*D*_*4*_	4	0.625
*S*_*7*_	*D*_*4*_	4	0.28452
*S*_*1*_	*D*_*5*_	5	0.7971
*S*_*2*_	*D*_*5*_	6	0.7569
*S*_*3*_	*D*_*5*_	8	0.79167
*S*_*4*_	*D*_*5*_	9	0.77222
*S*_*5*_	*D*_*5*_	8	0.87599
*S*_*6*_	*D*_*5*_	4	0.525
*S*_*7*_	*D*_*5*_	4	0.41667

For example, the data platform *D*_*2*_ wants to send a Mobile Agent to Platform *S*_*4*_. As described earlier, there is no entry for *S*_*4*_ with respect to *D*_*2*_. Hence, the Trust Lookup Table is traversed for other entries of *S*_*4*_ and the corresponding Trust Score. The extract of *S*_*4*_ from the Trust Lookup Table is as shown below in Table 2.

**Table 2:** Extract of server platform S_4_.

Server Platform	Data Platform	Transactions	Trust Score
*S*_*4*_	*D*_*1*_	9	0.77222
*S*_*4*_	*D*_*3*_	9	0.76794
*S*_*4*_	*D*_*4*_	9	0.77222
*S*_*4*_	*D*_*5*_	9	0.77222

In Table 2, it is visualised that there is no Trust Score Entry for *S*_*4*_ corresponding to *D*_*2*_. Therefore, in this case, the mean of the Trust Score is calculated. Now the Mean Trust Score for *S*_*4*_ is calculated as 0.77115. If the same service is provided by some other server platform for whom the Mean Trust Score is higher than that of *S*_*4*_, the Mobile Agent or the data server chooses the other server platform for the next migration.

Trust Monitor derives all the details relating to trust of a server platform with respect to a data platform. This information is collected from both Mobile Agent as well the server platform. The trust related data is obtained and computed for the server platforms only. It is because, these are the platforms which act as repository of data and are explored by the Mobile Agent for processing.

Each Mobile Agent is intercepted by the Trust Monitor to obtain the details pertaining to the various parameter related to Trust. Trust Monitor continuously monitors each and every transaction that happens within the system and stores the various parameters related to trust, performs calculations and stores it as Trust Score in the Trust Lookup Table. Trust Monitor maintains another table called the Trust Rank Table, which stores the Trustability Co-efficient of Variation (*V*) pertaining to each server platform and ranks them. Mobile Agents select the server platforms based on the ranks and plan the itinerary accordingly. The snapshot of the Trust Rank Table derived from the Trust Lookup Table is shown in Table 3.

**Table 3:** Snapshot of Trust Rank Table.

Server Platform	Trustability Co-efficient of Variation	Rank
*S*_*1*_	0.0001545367	2
*S*_*2*_	0.0024112654	5
*S*_*3*_	0.0004083333	3
*S*_*4*_	0.0000045918	1
*S*_*5*_	0.0026753258	6
*S*_*6*_	0.0022222222	4
*S*_*7*_	0.0038803855	7

**Fig 5:**
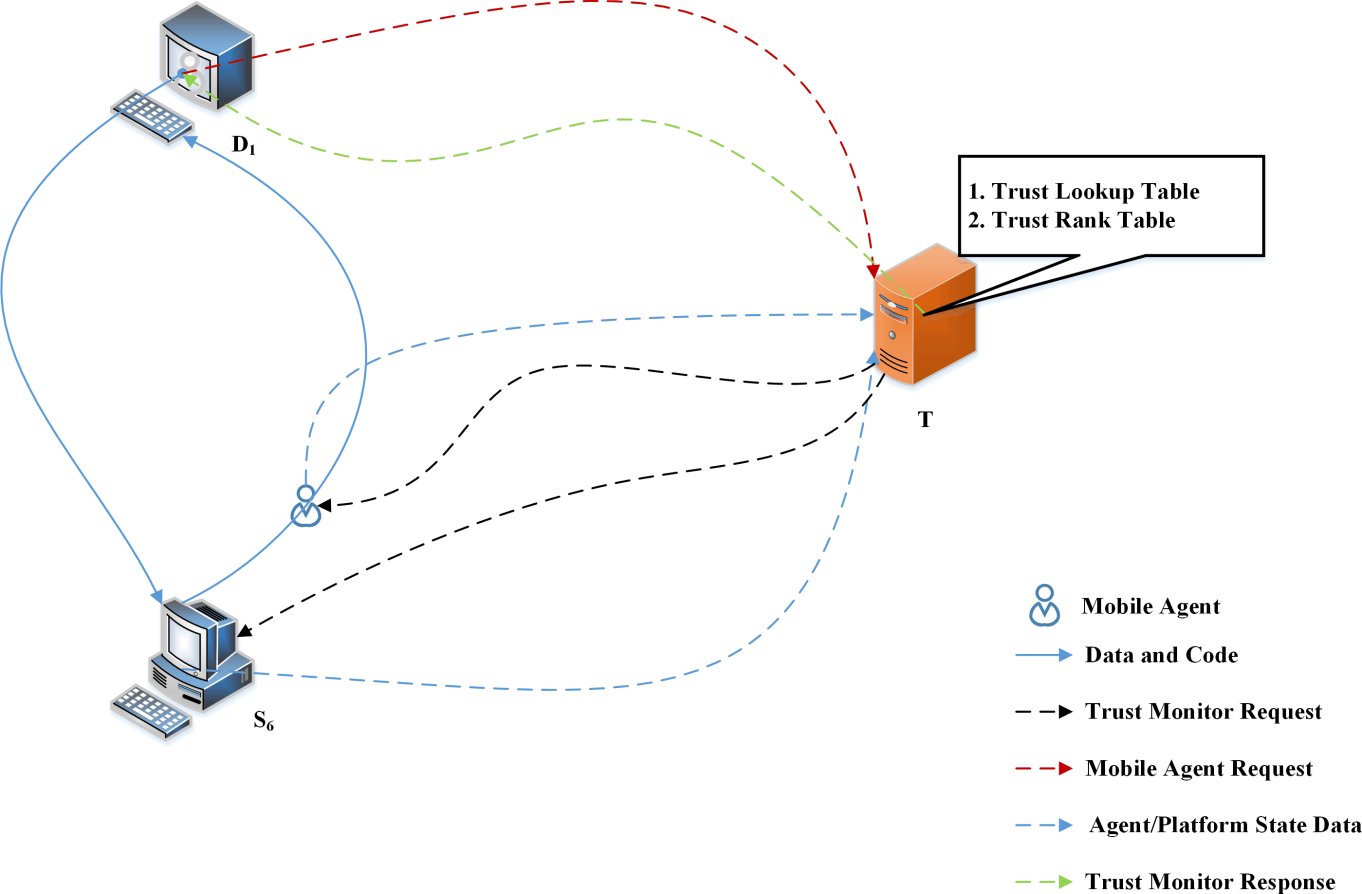
Communication between Mobile Agent, Server Platform and Trust Monitor.

The various transactions that happen between the Mobile Agent, server platform and the Trust Monitor are shown in Fig 5. Consider a Mobile Agent is initiated from the Data Platform *D*_*1*_, requires a data from a server platform. In this typical transaction, *S*_*6*_ is the server platform which processes the data. Before starting the itinerary, the Mobile Agent submits a request to the Trust Monitor (Mobile Agent Request in Fig 5), demanding the rank of the server platform towards which it has planned the itinerary. Once the Trust Monitor receives the Mobile Agent Request, it sends the rank of the server platform *S*_*6*_ (Trust Monitor Response in Fig 5). Along with this information, it also provides the rank of the server platforms providing the same type of service. The Mobile Agent decides the server platform to visit, based on the rank. If there are no other competent platforms or the Mobile Agent System is newly initiated (no trust based values will be available in the Trust Monitor), the Mobile Agent proceeds with the itinerary which it has planned. In this case, it immediately moves towards S_6_ for processing. Else, the Mobile Agent compares the ranks and plans the itinerary. In most cases, the Mobile Agent selects the server platform with the best rank.

Once the itinerary is initiated, the Mobile Agent moves from *D*_*1*_ to *S*_*6*_. If all the processes required by the Mobile Agent are completed. It moves back to *D*_*1*_. At this instance, Trust Monitor intercepts and collect various information relating to the particular transaction from the Mobile Agent and the server platform which are listed as follows:

- Provision/Denial of resources by the Server Platform *S*_*6*_ to the Mobile Agent
- Nature of request by the Mobile Agent (legitimate request or not)
- Turn-around time
- Response time
- Availability of information
- Rate of Synchronization
- Accuracy of Results and Information
- Usability of results of other platform, if any
- Degree of confidentiality

All the above mentioned parameters are gathered by the Trust Monitor for each transaction that occurs within the Mobile Agent System. These parameters are required by the Trust Monitor to calculate the Trust Score of a server platform, which in turn assists in the calculation of the Trustability Co-efficient of Variation of that server platform. At the time of calculation of the Trust Score of a specific platform, say *S*_*6*_, it is also possible to compute the Trust Score of some other platforms also. It is because, the usability of data provided by a platform can be endorsed by the other platform also. Therefore, at any moment of transaction, the Trust Monitor computes the Trust Score of more than one server platform.

### Performance analysis–response time

Trust Score based decision support system is compared with the reputation-based model for Mobile Agent migration for performance comparison. The reputation-based model for Mobile Agent migration utilizes the opinion-based belief structure and depends on seven parameters to decide the next hop of a Mobile Agent. Trust Score is based on five major factors, which in turn have nine major divisions. Though both the techniques are based on a number of factors, the decision making algorithm plays a vital role in the response time. The response time is the time taken by the data platform to communicate with the Trust Monitor for making the decision, in case of Trust Score or the time taken by the data platform to decide based on the opinion-based belief structure, in case of reputation-based model. The analysis is done using the experimental setup elaborated above.

The analysis is done in terms of average response time. The average response time for *m* mobile agents is calculated as follows:

$$\text{Average}\;\text{Response}\;\text{Time}\;=\frac{\sum_{n=1}^{m}T_{n}}{m}$$ (3)

where,

*T*_*n*_ is the response time incurred by the *n*^th^ Mobile Agent

*m* is the total number of Mobile Agents taken for observation

The observation, in terms of average response time is tabulated below in Table 4.

**Table 4:** Average Response Time comparison between reputation-based model, Trust Scoring System and Trust Ranking system.

No. of Mobile Agents	Average Response Time (in seconds)		
	Reputation-based Model	Trust Scoring System	Trust Ranking System
5	0.48	0.36	0.18
10	0.93	0.69	0.34
15	1.39	1.05	0.53
20	1.94	1.41	0.71
25	2.28	1.82	0.9
30	2.85	2.15	1.1
35	3.44	2.48	1.2
40	3.84	2.85	1.4
45	4.35	3.25	1.6
50	4.76	3.54	1.74
55	5.22	3.89	1.95
60	5.96	4.33	2.18
65	6.25	4.65	2.34
70	6.68	5.05	2.5
75	7.14	5.38	2.69
80	7.67	5.73	2.85
85	8.15	6.14	3.07
90	8.65	6.45	3.34
95	9.15	6.88	3.44
100	9.62	7.25	3.63
105	10.05	7.55	3.78
110	10.52	7.94	3.9
115	11.03	8.25	4.11
120	11.5	8.62	4.32
125	12.15	9.05	4.54
130	12.45	9.33	4.66
135	12.92	9.74	4.87
140	13.34	10.05	5.03
145	13.85	10.42	5.21
150	14.44	10.75	5.36
155	14.85	11.19	5.65
160	15.37	11.54	5.77
165	15.85	11.86	5.93

**Fig 6:**
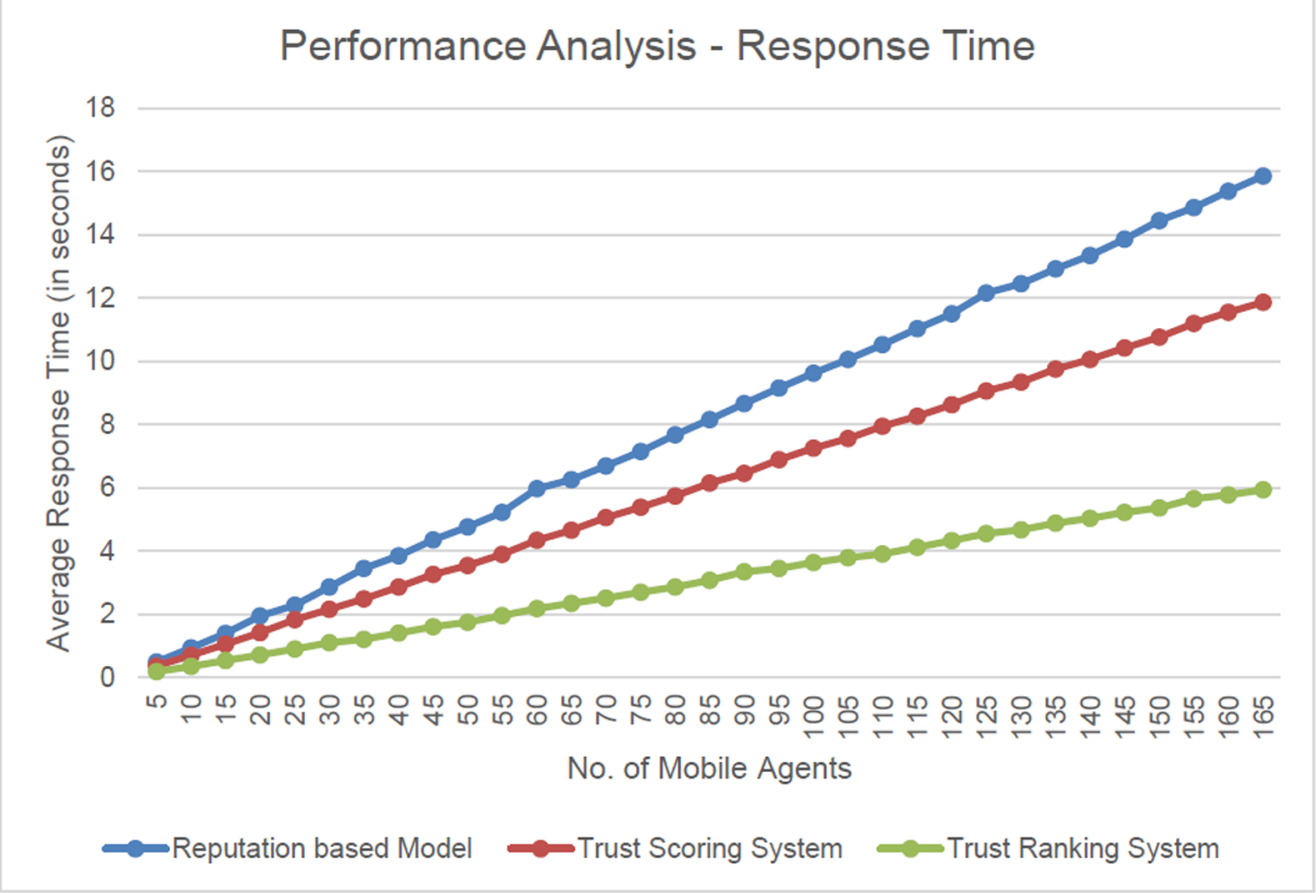
Performance analysis based on average response time.

The observations made are plotted and illustrated in Fig 6.

From Fig 6, it is observed that the Average Response Time taken in decision making in the Trust Scoring system is comparatively less compared to that of the reputation-based model.

It is observed that the Trust Scoring system, in an average consumes only 75% of the response time compared to that of the reputation-based model. Therefore, the Trust Scoring mechanism saves 25% of the time, in an average, taken for decision making compared to that of the reputation-based model.

It is inferred that the Trust Ranking system provides the faster response compared to that of the Trust Scoring system and reputation-based model.

It is because, in Trust Scoring system, the Mobile Agent needs to compute the average Trust Score based on the entries in the Trust Lookup Table. In case of Trust Ranking system, the overhead is balanced by the Trust Monitor itself. Since the Trust Monitor is dedicated for the storage of Trust Score alone, an additional responsibility of computing the Trustability Co-efficient of Variation and ranking server platforms accordingly is rendered to the Trust Monitor. This minimises the overall cost of computing which additionally contributed to the delay in the dispatch of the response in the Trust Scoring System. Therefore, the Trust Ranking system provides convincing results compared to that of the Trust Scoring system.

### Performance analysis–decision accuracy

The performance is analysed in terms of decision accuracy. Decision Accuracy may be defined as the rate of successful transactions made using a decision support system. The foremost objective of using a decision support system is to reduce the number of faulty transactions. Therefore, a decision support system like Trust Scoring system or reputation-based model need to be verified for accuracy in decision making to ensure the correctness of the system.

In such a perspective, performance analysis is made over the Trust Scoring system and the reputation-based model. A failure here, may be either due to non-responsive nature of the server platform, incompatibility, non-responsive nature of Mobile Agent, loss of Mobile Agent etc. In general, any transaction that ends up without producing any sort of result is termed a failure. The number of failures observed at different instance of time is tabulated in Table 5.

**Table 5:** Mobile Agent failure comparison between reputation-based model, Trust Scoring system and Trust Ranking system.

Total No. of Mobile Agents	No. of failed Mobile Agents		
	Trust Scoring System	Reputation-based Model	Trust Ranking System
5	1	1	0
11	2	3	0
19	3	5	0
28	4	6	1
36	5	7	1
40	7	9	2
44	10	13	4
49	11	15	5
58	14	19	7
68	15	22	8
77	16	26	9
82	17	28	10
88	19	30	11
96	20	34	12
105	21	36	13
113	23	40	14
117	24	42	15
121	27	43	17
126	28	45	18
132	29	46	19
140	30	49	20
149	31	53	21
157	32	55	22
161	34	58	23
165	36	60	24

**Fig 7:**
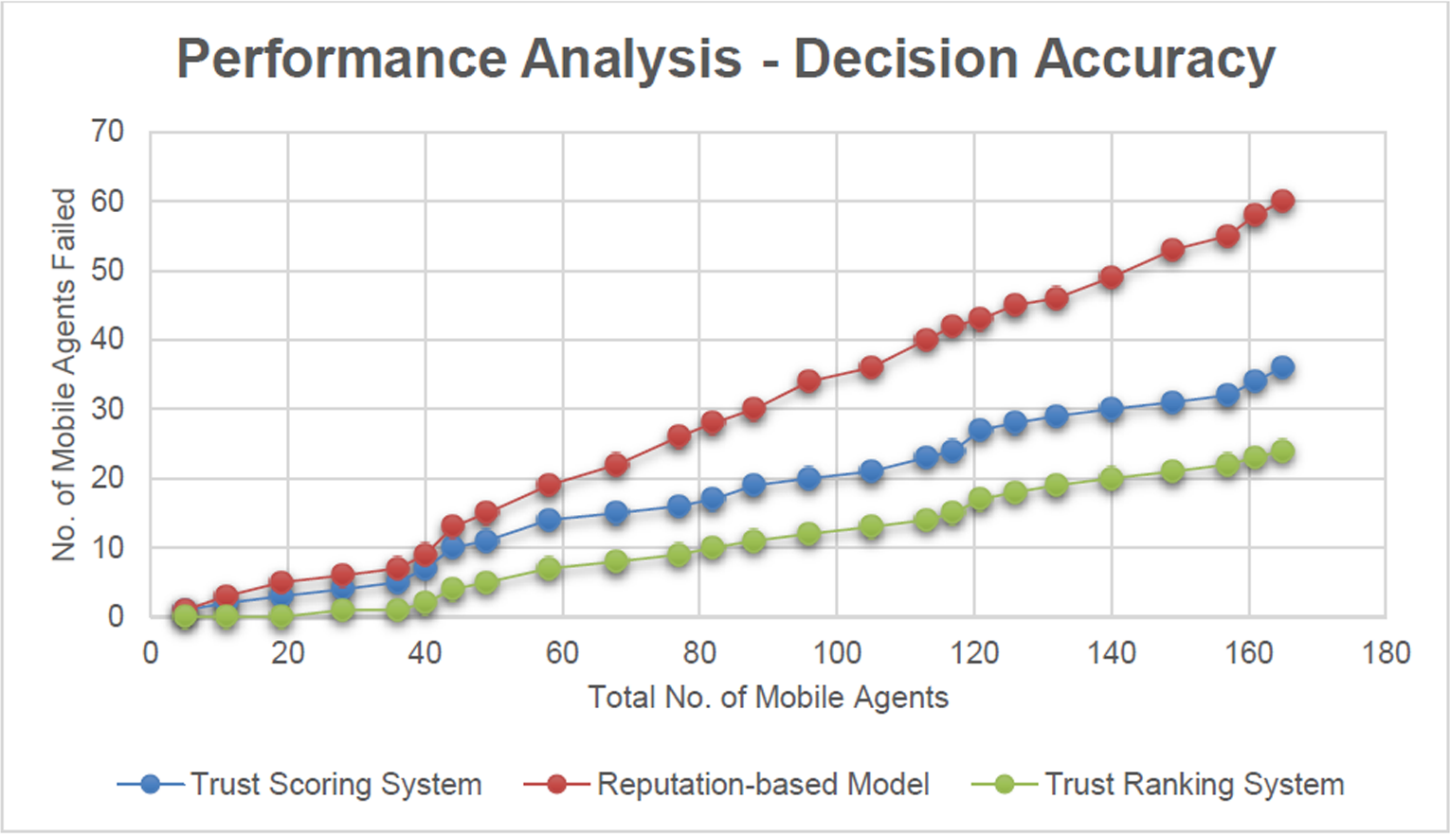
Performance analysis—decision accuracy (based on number of Mobile Agents failed).

The comparison is illustrated in terms of numbers in Fig 7.

**Fig 8:**
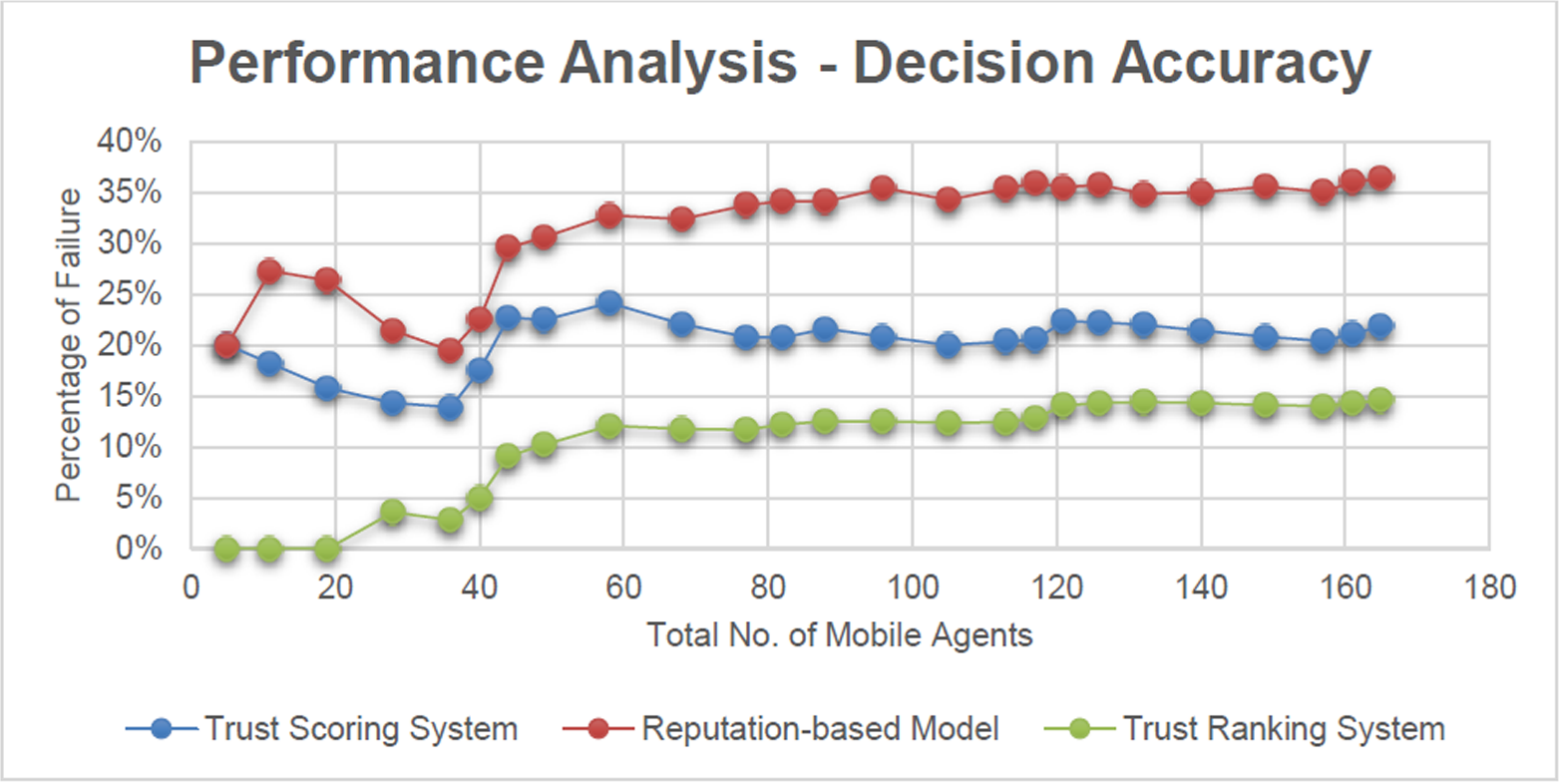
Performance analysis—decision accuracy (based on percentage of failure).

For a better understanding, the failure of Mobile Agents in terms of percentage is displayed in Fig 8.

From Fig 7, it is observed that the number of Mobile Agent failure is more in reputation-based model compared to that of the Trust Scoring System. This gets reflected in Fig 8, in which the comparison is taken in terms of percentage. The major drawback of the reputation based model is the fact that it concentrates less on compatibility, as a parameter.

It is observed that the success rate of Trust Scoring system is 80% whereas it is observed as 68% for the reputation-based model. This indicates that the Trust Scoring system provides accurate decisions 15% better than that of reputation based model.

It is observed that the Mobile Agents in the Trust Ranking System produces better results compared to that of the Mobile Agents in the Trust Scoring and reputation-based models.

This is explained by the fact that, the level of accuracy of the Trust Score is more refined when it is represented in terms of Co-efficient of Variation.

In Fig 8, a depiction of the failure of Mobile Agents in terms of percentages is displayed which gives a clear picture on the efficiency of the Trust Ranking System compared to that of the Trust Scoring System and reputation-based model. The percentage of failure is comparatively less in Trust Ranking System

In an average, the Trust Ranking System is 12.5% better than that of the Trust Scoring System. The efficiency described here is based on the inferences which are mentioned in the experimental setup described above.

### Advantages

The major advantage of using the Trust Ranking system compared to that of the Trust Scoring system is the reduction in computational overhead. In the Trust Scoring System, the Mobile Agent needs to traverse through the Trust Score entries in the Trust Lookup Table, calculate the mean to decide the itinerary. In Trust Ranking system, the Trust Rank Table contains the Trustability Co-efficient of Variation, which is an extract of the Trust Score entries in the Trust Lookup Table. Based on the Trustability Co-efficient of Variation, the ranking is done. The Mobile Agent needs to check the rank of the server platform, compare it with the rank of other server platforms providing the same service and determine the itinerary. This reduces the computation time by nearly 50%.

## Conclusion

The major contribution of this work is the introduction of Trust Score, a novel measure for assessing the trustworthiness of a platform and Trust Score based Itinerary planning Algorithm, which helps the Mobile Agent in decision-making based on Trust Score. The Trust Scoring system is enhanced by means of introducing Trustability Co-efficient of Variation. The Trust Scoring system produces multiple entries for the same server platform, but this system unifies the multiple entries using the Co-efficient of Variation of the entries which are unified as a new measure, Trustability Co-efficient of Variation. Based on the Trustability Co-efficient of Variation, the ranking is done for the server platforms. Based on experimental results, it is found that the proposed Trust Ranking system is better compared to that of the other existing decision support systems of that kind.

## Supporting information

S1 Appendix Tamil glossary data. (ZIP)
